# Immunophenotypic Characterization of Citrate-Containing A Concentrates in Maintenance Hemodialysis: A Pre-Post Study

**DOI:** 10.1155/2023/7772677

**Published:** 2023-09-27

**Authors:** Yuli Shen, Christoph Schmaderer, Andreas Ossadnik, Arianne Hammitzsch, Javier Carbajo-Lozoya, Quirin Bachmann, Vera Bonell, Matthias Christoph Braunisch, Uwe Heemann, Dang Pham, Stephan Kemmner, Georg Lorenz

**Affiliations:** ^1^Department of Nephrology, School of Medicine, Technical University of Munich, Klinikum Rechts der Isar, Munich, Germany; ^2^Nephrology and Rheumatology Department of the Second Affiliated Hospital, School of Medicine, The Chinese University of Hong Kong, Shenzhen & Longgang District People's Hospital of Shenzhen, Shenzhen 518172, China; ^3^German Centre for Infection Research (DZIF), Partner Site Munich, Munich, Germany

## Abstract

**Introduction:**

Due to chronic inflammation, maintenance hemodialysis (MHD) patients continue to show excess mortality. Acetate-free citrate-buffered A concentrates could be a way to improve the biocompatibility of the procedure, reduce chronic inflammation, and thus in the long term improve the prognosis of patients.

**Methods:**

Using a pre-post design (3 months of acetate followed by 3 months of citrate-acidified A concentrates in standard bicarbonate-based dialysate hemodialysis, CiaHD) and linear mixed model analysis in 61 stable HD patients, we assessed the impact of CiaHD on counts and phenotypes of peripheral T cells and monocytes by flow cytometry.

**Results:**

Switching to CiaHD left C-reactive protein (CRP) levels and leucocyte counts unaffected. However, CiaHD increased lymphocyte counts ex vivo. Furthermore, we found a decrease in total CD3+CD4+CD69+ ((10^9^/L), mean ± SD: acetate, 0.04 ± 1.0 versus citrate, 0.02 ± 0.01; *P* = 0.02) activated cells, while the number of CD28+ T cells remained stable. No differences were noted regarding T-cell exhaustion marker expression, CD14+CD16+ monocyte counts, and PMN-MDSCs.

**Conclusion:**

Compared with acetate, CiaHD has a minor impact on lymphocyte counts and CD4+T-cell activation, which was independent of systemic CRP and ionized magnesium, calcium levels, and other dialysis prescription modalities.

## 1. Introduction

Patients undergoing maintenance hemodialysis (MHD) have an adjusted mortality rate of 165 per 1000 patients/year [1]. This is partly attributed to a residual uremic environment and chronic inflammation [2, 3]. Although the pathophysiological mechanisms involved are not completely understood, immune dysfunction is regarded as a hallmark of progressive inflammation in MHD patients [4]. Chronic inflammation is known to increase the risk of infectious and cardiovascular diseases [5]. Immune dysfunction in MHD patients involves complex phenotypic changes in diverse immune cells [4, 6]. As a sign of aberrant T-cell activation, increased expression of the surface activation marker CD69 and loss of CD28 and a skewed CD4+/CD8+ ratio have been described [7, 8]. These alterations have been linked to systemic inflammation and atherosclerosis progression in MHD patients [9–11]. Others have reported lymphopenia in MHD patients with overexpression of PD-1 and TIM-3 on peripheral lymphocytes, indicating T-cell exhaustion and vulnerability to chronic infections and viral diseases [12, 13]. Furthermore, altered expression and interaction of costimulatory receptors such as CD28/PD1 with their ligands CD86/PDL1 might impact monocyte activation, resulting in innate immune system dysfunction [14, 15]. In this regard, the aberrant expansion of CD14+CD16+ monocytes has been identified as a predictor of mortality in HD patients [16, 17].

The spectrum of dysfunctional immune cells in MHD has recently been expanded for myeloid-derived suppressor cells (MDSCs). MDSCs can be divided into polymorphonuclear MDSCs (PMN-MDSCs) and monocytic MDSCs (M-MDSCs). Interestingly, PMN-MDSCs share similar phenotypical and morphological features with neutrophils and are increased in chronic infectious or inflammatory diseases [18, 19]. An expansion of PMN-MDSCs was reported for MHD patients and appeared to be associated with infections [20].

Acetate is widely adopted as an acidifying agent within the cation concentrate (A concentrate) in standard bicarbonate-based MHD. However, the intraindividual acetate concentration during HD procedures exceeds physiological levels, resulting in more intradialytic hypotensive episodes and associated adverse effects [21, 22]. In contrast, citrate-acidified A concentrates (CiaHD) in standard bicarbonate-based HD are considered more biocompatible. Compared to the conventional acetate-buffered HD procedure, an improvement in dialysis efficiency, a reduction of the systemic inflammatory response, i.e., serum C-reactive protein (CRP), and a better metabolic state have already been described by others [23–25]. Also, magnesium and calcium are essential cofactors required for immune cell activation [26]. Herein, citrate as a potent chelator of calcium and magnesium ions could lower free extracellular concentrations and potentially reduce aberrant immune cell activation [27]. However, the effect of citrate-buffered A concentrates in standard bicarbonate-based HD on the cellular immunophenotype is still unclear. This study was designed to investigate whether CiaHD impacts the cellular immunophenotypes in MHD patients.

## 2. Materials and Methods

### 2.1. Study Participants

Between April and June 2016, we recruited 78 MHD patients, of which 61 completed the entire study period. They were aged ≥18 years from two local dialysis units in Munich, Germany. All of them required thrice-weekly dialysis sessions with an average duration of ≥4 hours and HD vintages ≥two months. Missing data were due to unwillingness to complete the study, death, and technical reasons. Missing data were not imputed and referenced below the tables. Exclusion criteria were pregnancy, ongoing severe infection, cancer, malignant hematologic diseases, and lack of written or informed consent.

### 2.2. Study Design and Intervention

This post hoc pre-post design clinical trial was conducted in a subgroup of the “Substitution of Acetate by Citrate in Bicarbonate-Based-Hemodialysis” study (NCT02745340). Patients in two dialysis units from Munich were on acetate-containing A concentrates for 3 months (SelectBagOne; 3 mmol/L of acetate); they were then switched to three months of citrate-acidified A concentrates (SelectBagCitrate; acidified with citric acid, which is converted to 1 mmol/L citrate solution = CiaHD, supplied by Baxter). The clinical data and blood specimens were collected before (three months of acetate-containing A concentrates) and after three months of CiaHD prior to a midweek dialysis session as predefined in the mother study NCT02745340. It should be noted that centers were allowed to adjust the membrane and dialysis prescription to achieve adequate Kt/V or due to supply difficulties and fiscal reasons. The study protocol was approved by the local ethics commission. It was carried out in accordance with the Declaration of Helsinki, adhering to good clinical practice guidelines.

### 2.3. Clinical Data Assessment

Characteristics such as patients' age, gender, body mass index (BMI), comorbidities, and medication were assessed as previously described [24]. Immune suppressive mediation was recorded by chart review and included glucocorticoid, nonsteroidal anti-inflammatory drugs (NSAIDs), calcineurin inhibitor (tacrolimus in the study), and mycophenolate mofetil (MMF).

The cohort data set used to support the findings of this study has not been made available because of legal and patient privacy reasons—it is available from the corresponding author upon reasonable request in an anonymized form.

### 2.4. Blood Specimen Collection and Experimental Methods

Blood samples were collected prior to a midweek dialysis session prior and after three months of CiaHD and processed as previously described [24]. Ionized magnesium and calcium levels were determined from frozen sera using the NOVA 8 analyzer and ion-selective electrodes for calcium and magnesium following the manufacturer's instructions (Nova Biomedical, Waltham, MA, US) [28]. The number of neutrophils and lymphocytes and the level of CRP were examined by an ISO-certified clinical laboratory.

Peripheral blood mononuclear cells (PBMCs) were isolated using BD Vacutainer*®* CPT tubes within 2 hours post collection following the manufacturer's protocol. Cells were then stained and analyzed immediately using the antibodies provided in supplementary Table 1. FMOs were used to define the gating for CD28, CD69, and TIM-3, respectively. T cells were stained using antibodies for CCR7, CD25, CD45RO, CD3, CD4, CD8, CD28, CD69, PD1, and TIM3. Most surface markers were stained for 30 mins at 4°C. CCR7 was stained for 15 mins at 37°C before the following “cold” incubation with the remaining cocktail, including an additional washing step in between. PI was used as a viability dye. FSC-Height versus FSC-Width blots were used for the exclusion of doublets.

Monocytes were stained using CD3 and CD56 to exclude NK cells and T cells. CD14, CD16, HLA-DR-APC, and PDL1 were used to identify monocyte subpopulations. FMO was used to define gating for PDL1. Live/dead fixable blue stain (Thermo Fisher Scientific, Waltham, USA) was used as a viability dye.

PMN-MDCS was identified using CD14, CD15, and CD11b. Gating ancestries can be retrieved from supplementary Figures 1–3.

### 2.5. Statistical Analysis

SPSS Statistics 23 (IBM, Armonk, NY, USA) was used for statistical analysis. We report the percentage of total, mean ± standard deviation (SD), median, and interquartile range (IQR) as appropriate. The independent samples *t*-test and Wilcoxon–Mann–Whitney test were used for comparing the baseline data from different units as necessary. A linear mixed model was built to analyze the alterations of immune phenotypes in acetate versus CiaHD (pre-post design). Absolute counts of cell populations were examined as dependent variables, with treatment, (citrate = 1, acetate = 0) as the main effect. In addition, to check for confounding of our results, linear mixed models were adjusted for dialysis prescription characteristics, i.e. type of membrane, dialysis modality, vascular access, and dialysis session duration (see results section and supplement for further details). To test for confounding by hemoconcentration, intake of immunosuppressive drugs including NSAIDs, and dialysis modality (HD vs. hemodiafiltration (HDF)), an additional model was built and adjusted for these covariates (see supplementary Table 6).

Restricted maximum likelihood (REML) was used. *P* value <0.05 was considered significant. Since this was a post hoc exploratory study, no power calculation was performed.

## 3. Results

### 3.1. Study Population

Immunophenotypic data (pre-3 months of acetate and post-3 months of citrate treatment) were available for 61 patients. The mean age of participants was 69.9 (SD: ±15.6) years. 30 (49.2%) were male. The median HD vintage was 34 months, with 14 (23%) anuric patients (Table 1). Majority of patients (*n* = 59) were on hemodialysis (HD); two were prescribed hemofiltration (HDF). Arteriovenous fistulas were the preferred vascular access type (*n* = 53; 86.9%). The median modified Carlson comorbidity index [29] was 4 (2.8) in the study population, with arteriosclerosis (*n* = 22; 36.1%), mostly due to coronary heart disease (*n* = 20, 32.8%) and diabetes mellitus (*n* = 20, 32.8%) being the most frequent comorbidities (Table 1). Antihypertensives were the most frequent drugs prescribed (*n* = 58; 95.1%; Table 1), and 22 (36.1%) patients were on immunosuppressive medication including NSAIDs (*n* = 13; 21.3%), glucocorticoids (*n* = 7; 11.5%), calcineurin inhibitors (*n* = 1; 1.6%), or combined treatment (*n* = 1, 1.6%; Table 1). 15 (24.6%) patients had required hospitalization due to infectious events within 24 months before study inclusion. Patients from both dialysis units had a similar distribution of basal and laboratory characteristics (Table 1 and supplementary Table 2).

With regards to differences pre versus post CiaHD, it should be stated that one patient was switched from the AV fistula to the central venous catheter during the study course. Effective session duration was slightly shorter during CiaHD (acetate: 4.1 ± 0.2 vs. citrate: 3.9 ± 0.3 hours, *P* = 0.039, supplementary Table 3). In addition, different membranes were used over the course of the study in 46% of patients (supplementary Table 4), whereas other parameters of HD (F) prescription were kept constant as reported in supplementary Table 3. Nevertheless, our models were adjusted for these parameters to control for confounding (supplementary Table 5).

### 3.2. Citrate Neither Suppresses Systemic CRP Nor IL-6 Levels but Impacts Calcium and Magnesium Levels

**Figure 1:**
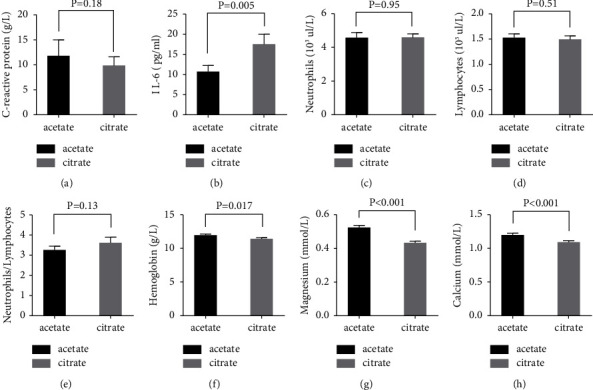
Alterations of clinical biomarkers before and after undergoing citrate-acidified A concentrates for three months. Histograms represent the mean with the standard error of mean. (a): C-reactive protein (patients: acetate, *n* = 59; citrate: *n* = 60), (b): IL-6 (patients: acetate, *n* = 58; citrate: *n* = 58), (c): neutrophils (patients: acetate, *n* = 60; citrate: *n* = 61), (d): lymphocytes (patients: acetate, *n* = 61; citrate: *n* = 61), (e): neutrophils/lymphocytes (patients: acetate, *n* = 60; citrate: *n* = 61), (f): hemoglobin (patients: acetate, *n* = 59; citrate: *n* = 60), (g): magnesium (patients: acetate, *n* = 61; citrate: *n* = 59), and (h): calcium (patients: acetate, *n* = 61; citrate: *n* = 59). *P* = 0.05 was considered significant due to the exploratory design of the study.

After three months of CiaHD, similar systemic CRP levels ((g/l), mean ± SD: acetate, 11.8 ± 24.4 versus citrate, 9.9 ± 13.4; *P* = 0.18) were recorded (Table 2, Figure 1(a)). Contrary to our expectations, a slight increase in systemic IL-6 levels ((pg/ml), mean ± SD: acetate, 10.5 ± 11.7 versus citrate, 17.5 ± 18.8; *P* = 0.005) was detected (Table 2, Figure 1(b)). In line with CRP, CiaHD had little impact on the number of neutrophils, lymphocytes, and NLR (mean ± SD: acetate, 3.3 ± 1.5 versus citrate, 3.6 ± 2.3; *P* = 0.13) (Table 2, Figures 1(c)–1(e)). As expected, citrate did significantly reduce systemic ionized magnesium ((mmol/L), mean ± SD: acetate, 0.5 ± 0.1 versus citrate, 0.4 ± 0.08; *P* < 0.001) and calcium levels ((mmol/L), mean ± SD: acetate, 1.2 ± 0.2 versus citrate, 1.1 ± 0.2; *P* < 0.001) (Table 2, Figures 1(g) and 1(h)).

### 3.3. Citrate-Containing A Concentrates Are Associated with Reduced Activation of CD4+ T Cells

Still, due to various reports by others [13–16], the initial hypothesis was that CiaHD would beneficially impact the chronic inflammatory milieu and alter the T-cellular inflammatory phenotypes, which could still occur in the absence of measurable alterations in systemic CRP levels.

**Figure 2:**
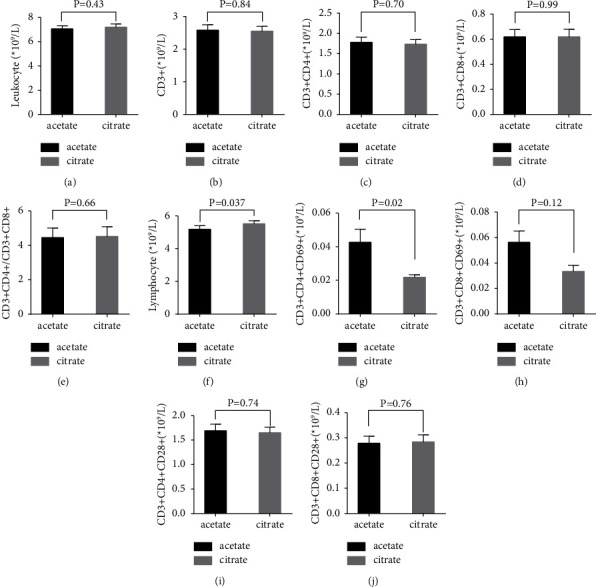
Absolute numbers of leucocytes (a) and T-lymphocyte phenotypes (b‐j) before and after citrate‐acidifed A concentrate dialysis for three months. Histograms present means with the standard error mean for 61 patients for (b) CD3 + cells, (c) CD3 + CD4 + T cells (d) CD3 + CD8 + Tcells, (e) CD3 + CD4/CD8 ratios, (f) Lymphocyte counts (g) CD3 + CD4 + CD69 + activated T cells, (h) CD3 + CD8 + CD69 activated T cells, (i) CD3 + CD4 + CD28 + activated T cells, (j) CD3 + CD8 + CD28 + activated T cells, respectively. P < 0.05 was considered significant due to the exploratory design of the study.

**Figure 3:**
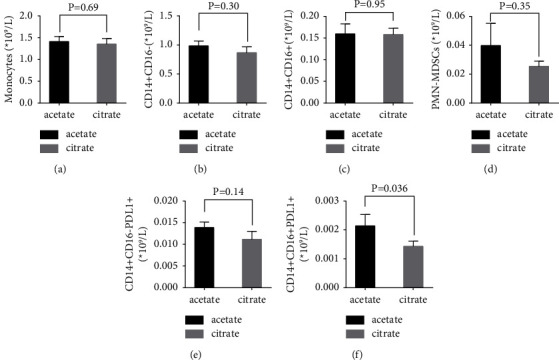
The alterations of monocytes and the subset PMN-MDSC and PDL1+ cells before and after switching to citrate-acidified A concentrate dialysis for three months. Absolute cell numbers are presented as histograms (mean with the standard error of mean). (a): Monocytes, (b): CD14+CD16−, (c): CD14+CD16+, (d): PMN-MDSCs (patients: acetate, *n* = 52; citrate, *n* = 61), (e): CD14+CD16−PDL1+, and (f): CD14+CD16−PDL1+ (patients: acetate, *n* = 61; citrate, *n* = 60). *P* = 0.05 was considered significant due to the exploratory design of the study.

The absolute leucocyte and overall CD3+, CD3+CD4+, and CD3+CD8+ cell numbers were not affected by CiaHD (Table 3, Figures 2(a)–2(d)). The CD3+CD4+/CD3+CD8+ ratio remained unchanged (*P* = 0.60) (Table 3, Figure 2(e)), whereas lymphocytes slightly increased after CiaHD (Table 3, Figure 3(f)).

However, we found slightly decreased numbers of “activated” CD3+CD4+CD69+ T cells ((10^9^/L), mean ± SD: acetate, 0.04 ± 1.0 versus citrate, 0.02 ± 0.01; *P* = 0.02) pre- (after 3 months of standard acetate A concentrates) versus post-3 months of citrate A concentrates. CD3+CD4+CD69+ cells were significantly associated with the treatment (citrate = 1) in the linear mixed model analysis (*β* = −0.02, *P* values 0.02, Table 3, Figure 2(g)). This was further observed after adjusting the model for changes in dialysis membranes, effective session duration, vascular access, and dialysis modality (*β* = −0.02, *P* value 0.02; supplementary Table 5).

Similarly, the counts of CD3+CD8+CD69+ cells tended to decrease (*β* = −0.01, *P* value 0.12), whereas CD3+CD4+CD28+ and CD3+CD8+CD28+ subsets remained stable on acetate versus citrate A concentrates (Table 3, Figures 2(h)–2(j)), when the model was adjusted for change in membranes, effective session duration, vascular access type, and HD modality (supplementary Table 5).

It should be noted that there was no significant interaction effect of CRP treatment (citrate = 1) on CD3+CD4+CD69+ cells. Furthermore, calcium and magnesium levels were inversely associated with these cells and did not significantly improve the regression model (Table 4).

Analysis of exhaustion markers, PD-1 and TIM-3, on peripheral lymphocytes did not reveal altered frequencies of PD1+ T cells. Yet, a trend of slightly lower numbers of TIM3+CD3+CD8+ T cells was associated with 3 months of citrate-buffered A concentrates ((10^9^/L), mean ± SD: acetate, 0.007 ± 0.005 versus citrate, 0.006 ± 0.005; *P* = 0.21) (Table 3, supplementary Figure 4(A)–4(D)). Therefore, we conclude that citrate might impact T-cell activation and exhaustion status in MHD patients in the absence of measurable alterations in systemic CRP levels.

### 3.4. Acetate-Free Citrate-Containing Dialysis Solutions Leave Monocyte Populations Unaffected

Next, monocyte subtypes were analyzed in a similar fashion. CiaHD did not affect the overall count of monocytes (Table 5, Figure 3(a)). CD14+CD16− ((10^9^/L), mean ± SD: acetate, 1.0 ± 0.7 versus citrate, 0.9 ± 0.8; *P* = 0.30) and CD14+CD16+ ((10^9^/L), mean ± SD: acetate, 0.2 ± 0.2 versus citrate, 0.2 ± 0.1; *P* = 0.95) (Table 5, Figures 3(b) and 3(c)) subsets and pathologically activated neutrophils and monocytes with immunosuppressive capacities known to be expanded and linked to infectious events in HD patients a.k.a [20] PMN-MDSCs ((10^9^/L), mean ± SD: acetate, 0.04 ± 0.1 versus citrate 0.03 ± 0.3; *P* = 0.35) remained unchanged (Table 4, Figure 3(d)). Similarly, we did not find significant alterations in numbers of PDL1+ CD14+CD16− cells after 3 months of citrate treatment (Table 5, Figure 3(e)). A mild decrease of PDL1+ CD14+CD16+ cells was found after CiaHD ((10^9^/L), mean ± SD: acetate, 0.002 ± 0.003 versus citrate 0.001 ± 0.01; *P* = 0.036) (Table 5, Figure 3(f)). However, statistical significance of this finding was lost when the model was adjusted for change in membranes, effective session duration, vascular access type, and HD modality (*P* value = 0.2; supplementary Table 5).

## 4. Discussion

Chronic inflammation and aberrant immune cell activation are well described characteristics of MHD patients. All of them have been linked to adverse outcomes in this population [30–32]. Compared with acetic-acid-buffered A concentrates in standard bicarbonate HD, CiaHD has been reported to enhance HD efficiency and reduce systemic inflammation and vascular smooth muscular cell (VSMC) dysfunction [33, 34]. Furthermore, CiaHD has been one attempt to improve the HD-procedure's biocompatibility and restore immunity in end-stage renal disease (ESRD) [23, 35]. Nevertheless, and in contrast to similar studies [33, 36], in our pre-post study, CiaHD did not beneficially influence systemic CRP or IL-6 levels. However, our data are not unique in finding stable or increasing systemic CRP and IL-6 levels [35], which might be related to patient individual factors, differently tuned dialysate mixtures, or cohort size.

Despite that, we found a significant reduction of circulating activated CD3+CD4+CD69+ cells after 3 months of CiaHD, indicating some impact on cellular immunity. Still, the post hoc and exploratory study design must be considered when interpreting these data. In our cohort, reduction of CD3+CD4+CD69+ cells during CiaHD treatment occurred independently of changes in CRP and systemic magnesium and calcium levels, which in contrast to CiaHD were not significantly associated with CD3+CD4+CD69+ cells (Table 4). This association was further independent of different HD-membranes used in 46% of study participants and other parameter prescription. Thus, citrate per se (independent of its function as a chelator) or the lack of supra-physiological acetate concentrations might have reduced CD4+ T-cell activation. In fact, acetate has been shown to impact T-cell effector function and promote interferon production upon chronic inflammatory conditions such as tumor environments [37]. These considerations however remain speculative and require further study.

In addition, and consistent with previous studies [38], we found increased lymphocyte counts after 3 months of CiaHD. T-cell lymphopenia in hemodialysis is documented to increase the risk of infectious episodes and is considered a marker of impaired immunity [13, 39–41]. Taken together, it is tempting to speculate that CiaHD could reverse some aspects of aberrant immunity in MHD, namely, CD4+ T-cell activation and lymphopenia. Yet, the overall impact of CiaHD on immunophenotypic changes appeared small, as CD14+CD16+ monocyte counts and the number of PMN-MDSCs, which are known to suppress T-cell activation and function during infectious conditions [42], were unaffected, and no beneficial serologic changes were noted. Lastly, it should be mentioned that different membrane types matched by dialysis centers were also independently associated with a reduction in CD4+CD69+ T cells (see supplementary Table 5). Thus, cumulatively, further optimization of MHD biocompatibility seems feasible and reasonable by several approaches.

Taken together, our study is merely a characterization of immune-phenotypic changes related to acetate-free citrate-acidified A concentrates in standard bicarbonate MHD. Several limitations must be mentioned. Since this was an exploratory and descriptive study with only a decent sample size, we cannot exclude residual confounding including nonobserved changes in dialysis prescription by the participating units. In addition, the immune-phenotypic changes observed were relatively minor on citrate versus acetate-containing dialysates with no effect on the systemic proinflammatory mediator milieu. Furthermore, we lacked prospective data on clinical endpoints to define the clinical implications of the observed immune-phenotypic changes.

In conclusion, compared with acetate-containing A concentrates in standard bicarbonate MHD, CiaHD had a mild impact on the cellular immunophenotype: increased lymphocyte count and reduced CD3+CD4+ T-cell activation, indicating that CiaHD could revert some disorders of cellular immunity independently of systemic CRP levels. Investigating the implications of these changes requires more extensive studies.

## Figures and Tables

**Table 1:** Baseline characteristics of the study population.

Parameters	Total *N* = 61	Unit 1 *N* = 35	Unit 2 *N* = 26	*P* value
Age (years; mean ± SD)	69.9 ± 15.6	69.6 ± 16.6	70.2 ± 14.6	0.90
Gender, male (*n*%)	30 (49.2)	19 (54.3)	11 (42.3)	0.36
BMI (kg/m^2^, mean ± SD)	25.8 ± 4.8	25.9 ± 5.0	25.7 ± 4.6	0.86
Dialysis vintage (months; median, IQR)	34 (15.59)	38 (17.58)	29 (14.61)	0.67
Anuric (*n*%)	14 (23.0)	5 (14.3)	9 (34.6)	0.06
Overweight (*n*%)	30 (49.2)	16 (45.7)	14 (53.8)	0.53
CCI	4 (2.8)	6 (3.8)	2.5 (1.7)	0.16
Arteriosclerosis (*n*%)	22 (36.1)	15 (42.9)	7 (26.9)	0.20
Coronary heart disease (*n*%)	20 (32.8)	13 (37.1)	7 (26.9)	0.40
Diabetes mellitus (*n*%)	20 (32.8)	13 (37.1)	7 (26.9)	0.40
History of myocardial infarction (*n*%)	11 (18.0)	7 (20)	4 (15.4)	0.65
Central vascular disease (*n*%)	12 (19.7)	7 (20)	5 (19.2)	0.94
Heart failure (*n*%)	5 (8.2)	3 (8.6)	2 (7.7)	0.90
Pulmonary hypertension (*n*%)	7 (11.5)	5 (14.3)	2 (7.7)	0.43
Atrial fibrillation (*n*%)	16 (26.2)	10 (28.6)	6 (23.1)	0.63
COPD (*n*%)	6 (9.8)	6 (17.1)	0 (0)	0.03
Peripheral arterial disease (*n*%)	11 (18)	7 (20.0)	4 (15.4)	0.65
Medication				
Immunosuppressive medication (*n*%)	22 (36.1)	11 (31.4)	11 (42.3)	0.39
Glucocorticoid (GC)	7 (11.5)	1 (2.9)	6 (23.1)	
NSAIDs	13 (21.3)	9 (25.7)	4 (15.4)	
Tacrolimus	1 (1.6)	1 (2.9)	0	
Mycophenolate mofetil	1 (1.6)	0	1 (3.8)	
GC + MMF + NSAID	1 (1.6)	0	1 (3.8)	
Statin (*n*%)	29 (47.5)	17 (48.6)	12 (46.2)	0.85
Blood pressure medication (*n*%)	58 (95.1)	33 (94.3)	25 (96.2)	0.74
Patients required hospitalization due to infectious events within 24 months^*b*^	15 (24.6)	12 (34.3)	3 (11.5)	0.05

Body mass index (BMI); Carlson comorbidity index (CCI); chronic obstructive pulmonary disease (COPD); nonsteroidal anti-inflammatory drugs (NSAIDs). Values are given as either mean ± standard deviation or number (percentage) or median (IQR). The independent-samples *t*-test and the Wilcoxon–Mann–Whitney *U* test were used for comparison of dialysis units at baseline. *P* < 0.05 was considered significant. ^*a*^One patient was prescribed combined immunosuppressive treatment. ^*b*^1 missing value.

**Table 2:** Changes in clinical parameters after switching to citrate-acidified A concentrates.

Parameter	Treatment (mean ± SD)		Linear mixed model	
			Main effect: treatment	
	Acetate	Citrate	Estimate (*β*)	*P* value
C-reactive protein (g/L)	11.8 ± 24.4	9.9 ± 13.4	−2.8	0.18
IL-6 (pg/ml)	10.5 ± 11.7	17.5 ± 18.8	7.0	0.005
Neutrophils (10^3/*μ*l)	4.6 ± 2.4	4.6 ± 1.5	−0.02	0.95
Lymphocytes (10^3/*μ*l)	1.5 ± 0.6	1.5 ± 0.5	−0.03	0.51
Neutrophils/lymphocytes	3.3 ± 1.5	3.6 ± 2.3	0.37	0.13
Hemoglobin (g/L)	12.0 ± 1.3	11.4 ± 1.3	−0.52	0.017
Magnesium (mmol/L)	0.5 ± 0.1	0.4 ± 0.08	−0.09	<0.001
Calcium (mmol/L)	1.2 ± 0.2	1.1 ± 0.2	−0.10	<0.001

Interleukin6 (IL-6). A linear mixed model was built to analyze the parameters before and after switching to citrate-acidified A concentrates (with treatment as the main effect: citrate = 1, acetate = 0). *P* < 0.05 was considered significant. Missing values were as follows: in acetate treatment, C-reactive protein/hemoglobin, *n* = 2; IL-6, *n* = 3; neutrophils, *n* = 1; neutrophils/lymphocytes, *n* = 1; in citrate treatment, C-reactive protein, *n* = 1; IL-6, *n* = 3; magnesium/calcium, *n* = 2.

**Table 3:** Changes in leucocyte and T-cell phenotypes after switching to citrate-acidified A concentrates.

Phenotypes (10^9^/L)	Treatment (mean ± SD)		Linear mixed model	
			Main effect: treatment	
	Acetate	Citrate	Estimate (*β*)	*P* value
Leucocyte^*b*^	7.1 ± 2.0	7.2 ± 2.0	−0.13	0.43
Lymphocyte	5.2 ± 1.7	5.5 ± 1.4	−0.33	0.037
CD3+	2.6 ± 1.3	2.6 ± 1.2	0.03	0.84
CD3+CD4+	1.8 ± 1.0	1.7 ± 0.9	−0.05	0.70
CD3+CD8+	0.6 ± 0.4	0.6 ± 0.5	0.0007	0.99
CD3+CD4+/CD3+CD8+	4.4 ± 4.4	4.5 ± 4.3	−0.076	0.66
CD3+CD4+CD69+	0.04 ± 0.1	0.02 ± 0.01	−0.02	0.02
CD3+CD8+CD69+	0.05 ± 0.07	0.04 ± 0.04	−0.01	0.12
CD3+CD4+CD28+	1.7 ± 1.0	1.7 ± 0.9	0.04	0.74
CD3+CD8+CD28+	0.3 ± 0.2	0.3 ± 0.2	−0.005	0.76
CD3+CD4+PD1+	0.6 ± 0.3	0.6 ± 0.3	−0.04	0.29
CD3+CD8+PD1+	0.2 ± 0.2	0.2 ± 0.2	−0.001	0.93
CD3+CD4+TIM3+	0.03 ± 0.02	0.03 ± 0.02	<0.001	0.99
CD3+CD8+TIM3+	0.007 ± 0.005	0.006 ± 0.005	0.0009	0.21

A linear mixed model was built to analyze the parameters before and after switching to citrate-acidified A concentrates (with treatment as the main effect: citrate = 1, acetate = 0). To test for robustness of our results and to rule out confounding as far as possible, another model was built and adjusted for: HD-membranes used during acetate versus citrate, vascular access type, HD vs. HD (F), and session duration (see supplementary Table 5). Similar results as depicted above were obtained for treatment (citrate = 1). *P* < 0.05 was considered significant.

**Table 4:** Regression model of CD4+CD69+ with CRP, Mg, and Ca.

	Unstandardized coefficients	Std. error	Standardized coefficients	*T*	Sig.	VIF
(Constant)	0.006	0.025		0.227	0.82	
CRP	0	0	0.098	1.037	0.30	1.028
Mg	−0.012	0.047	−0.029	−0.258	0.80	1.482
Ca	0.025	0.024	0.118	1.04	0.30	1.493
Dependent variable: CD4+/CD69+						

C-reactive protein (CRP); magnesium (Mg); calcium (Ca). The regression model was built to analyze the relationship of CD4+CD69+ with CRP, Mg, and Ca. *P* < 0.05 was considered significant.

**Table 5:** Changes in monocyte phenotypes and PMN-MDSCs after switching to citrate-acidified A concentrates.

Monocyte and PMN-MDSC phenotype (10^9^/L)	Treatment (mean ± SD)		Linear mixed model	
			Main effect: treatment	
	Acetate	Citrate	Estimate (*β*)	*P* value
Monocyte^*a*^	1.4 ± 0.9	1.4 ± 1.0	−0.06	0.69
CD14+CD16−^*a*^	1.0 ± 0.7	0.9 ± 0.8	−0.12	0.30
CD14+CD16+^*a*^	0.2 ± 0.2	0.2 ± 0.1	−0.001	0.95
CD14−CD15+ PMN−MDSC^*b*^	0.04 ± 0.1	0.03 ± 0.3	−0.013	0.35
CD14+CD16−PDL1+^*a*^	0.01 ± 0.01	0.01 ± 0.01	−0.003	0.14
CD14+CD16+PDL1+^*a*^	0.002 ± 0.003	0.001 ± 0.001	−0.0008	0.036

A linear mixed model was built to analyze the parameters before and after switching to citrate dialysate (with treatment as the main effect: citrate = 1, acetate = 0). To test for robustness of our results and to rule out confounding as far as possible, another model was built and adjusted for: HD-membranes used during acetate versus citrate, vascular access type, HD vs. HD (F), and session duration (see supplementary Table 4). Similar results as depicted above were obtained for treatment (citrate = 1). *P* < 0.05 was considered significant. ^*a*^1 missing values in the citrate treatment group. ^*b*^9 missing values of PMN-MDSCs in acetate treatment groups.

## Data Availability

The data used to support the findings of this study are available from the corresponding author upon reasonable request.

## Supplementary Materials

Supplementary Materials Supplementary Figure 1: A–N: the gating ancestry of T cells from peripheral blood mononuclear cells isolated by CPT® gradient centrifugation ex vivo. Cells were gated as displayed in A–E to isolate CD3+ cells. F–J: applied for surface marker gating on CD3+CD4+cells. A similar approach was applied for CD3+CD8+ cells (K–N). Supplementary Figure 2: the gating strategy of monocytes from peripheral blood mononuclear cells isolated by CPT® gradient centrifugation ex vivo. A–H: selection of CD14+/CD16 ± monocytes. I–J: defining PDL+1 cells amongst CD14+CD16 ± cells, respectively. Supplementary Figure 3: A–F: gating strategy of MDSCs from peripheral blood mononuclear cells isolated by CPT® gradient centrifugation ex vivo. Supplementary Figure 4: alterations of T-cell exhaustion markers before and after undergoing acetate or citrate-buffered A concentrates for three months, respectively. The number of phenotypes is presented as histograms (mean and standard error of mean) for 61 patients. Level of significance *P* < 0.05. Supplementary Table 1: monoclonal antibodies used for flow cytometry. Supplementary Table 2: dialysis characteristics and dialysate compositions at baseline. Supplementary Table 3: changes of dialysis prescription, filters, and dialysis modality per treatment period (acetate vs. citrate). Supplementary Table 4: use of dialysis membranes during acetate and citrate A concentrates. Supplementary Table 5: linear mixed model analysis, adjusted for changes in filters, session duration, and dialysis modality per treatment period with treatment as a main effect and cellular phenotypes as dependent variables. Supplementary Table 6: linear mixed model analysis, adjusted for changes in filtration volume, immunosupression medication, and dialysis modality per treatment period with treatment as a main effect and cellular phenotypes as dependent variables.
