# Supplementary material for: Immunophenotypic Characterization of Citrate-Containing A Concentrates in Maintenance Hemodialysis: A Pre-Post Study
Source: Int J Nephrol. 2023 Sep 27;2023:7772677. doi: 10.1155/2023/7772677 (PMC10551471; doi:10.1155/2023/7772677)
Supplement: Supplementary Materials — Supplementary Figure 1: A–N: the gating ancestry of T cells from peripheral blood mononuclear cells isolated by CPT® gradient centrifugation ex vivo. Cells were gated as displayed in A–E to isolate CD3+ cells. F–J: applied for surface marker gating on CD3+CD4+cells. A similar approach was applied for CD3+CD8+ cells (K–N). Supplementary Figure 2: the gating strategy of monocytes from peripheral blood mononuclear cells isolated by CPT® gradient centrifugation ex vivo. A–H: selection of CD14+/CD16 ± monocytes. I–J: defining PDL+1 cells amongst CD14+CD16 ± cells, respectively. Supplementary Figure 3: A–F: gating strategy of MDSCs from peripheral blood mononuclear cells isolated by CPT® gradient centrifugation ex vivo. Supplementary Figure 4: alterations of T-cell exhaustion markers before and after undergoing acetate or citrate-buffered A concentrates for three months, respectively. The number of phenotypes is presented as histograms (mean and standard error of mean) for 61 patients. Level of significance P < 0.05. Supplementary Table 1: monoclonal antibodies used for flow cytometry. Supplementary Table 2: dialysis characteristics and dialysate compositions at baseline. Supplementary Table 3: changes of dialysis prescription, filters, and dialysis modality per treatment period (acetate vs. citrate). Supplementary Table 4: use of dialysis membranes during acetate and citrate A concentrates. Supplementary Table 5: linear mixed model analysis, adjusted for changes in filters, session duration, and dialysis modality per treatment period with treatment as a main effect and cellular phenotypes as dependent variables. Supplementary Table 6: linear mixed model analysis, adjusted for changes in filtration volume, immunosupression medication, and dialysis modality per treatment period with treatment as a main effect and cellular phenotypes as dependent variables. [file 7772677.f1.zip › 2306_Supplement_CiAc_T_Cell_RESUBM_V3.docx]

**Supplementary tables: Immunophenotypic characterization of citrate containing A concentrates in maintenance hemodialysis – a pre-post study.**

**Supplementary table 1**. Monoclonal Antibodies used for flow cytometry

| **Application** | **Target antigen/** **fluorescence** | **Company** |
| --- | --- | --- |
| Viability dye | Propidium Iodide | Sigma Aldrich, St. Louis, USA |
|  | Live/Dead Fixable Blue Stain | Thermo Fisher Scientific, Waltham, USA |
| T cells surface antigens | Anti-Human CD25 PE-Dazzle | Biolegend, San Diego, USA |
|  | Anti-Human CCR7 FITC | Biolegend, San Diego, USA |
|  | Anti-HumanCD45RO PerCP/Cy5.5 | Biolegend, San Diego, USA |
|  | Anti- Human CD3 BV605 | Biolegend, San Diego, USA |
|  | Anti- Human CD4 BV785 | Biolegend, San Diego, USA |
|  | Anti- Human CD8 BV510 | Biolegend, San Diego, USA |
|  | Anti- Human CD69 AlexaFluor700 | Biolegend, San Diego, USA |
|  | Anti- Human CD28 APC | Biolegend, San Diego, USA |
|  | Anti- Human PD-1 PE | Biolegend, San Diego, USA |
|  | Anti- Human Tim3 BV421 | Biolegend, San Diego, USA |
| Monocytes surface antigens | Anti- Human CD56 BV785 | Biolegend, San Diego, USA |
|  | Anti- Human CD3 BV605 | Biolegend, San Diego, USA |
|  | Anti- Human HLA-DR APC | Biolegend, San Diego, USA |
|  | Anti- Human CD14 PerCP/Cy5.5 | Biolegend, San Diego, USA |
|  | Anti-Human CD16 FITC | Biolegend, San Diego, USA |
|  | Anti-Human PDL-1 BV510 | BD Bioscience, San Jose, USA |
| PMN-MDSCs | Anti- Human CD14 APC/Cy7 | Biolegend, San Diego, USA |
|  | Anti-Human CD15 Pacific Blue | Biolegend, San Diego, USA |
|  | Anti-HumanCD11b AlexaFluor700 | Biolegend, San Diego, USA |

Abbreviations: polymorphonuclear Myeloid-derived suppressor cells (PMN-MDSCs); T cells were stained using antibodies for CD25, CD45RO, CD3, CD4, CD8, CD28, CD69, PD1, and TIM3. Monocytes were stained using CD3 and CD56 to exclude NK cells, and T cells surface markers were stained for CD14, CD16, HLA-DR-APC and PDL1 to identify monocyte subpopulations; PMN-MDSCs were identified using CD14, CD15, and CD11b. All surface markers except for CCR7 were stained for 30mins at 4°C.

**Supplementary table 2:** Dialysis characteristics and dialysate compositions at baseline

| **Dialysis information** | **Total**  **N=61** | **Unit 1**  **N=35** | **Unit 2**  **N=26** | **P-value** |
| --- | --- | --- | --- | --- |
| Dialysis Vascular Access(n%) |  |  |  | 0.23 |
| Arterio-Venous Fistula | 53(86.9) | 32(91.4) | 21(80.8) |  |
| Arterio-Venous Graft | 3(4.9) | 1(2.9) | 2(7.7) |  |
| Catheter | 5(8.2) | 2(5.7) | 3(11.5) |  |
| Filter(n%) |  |  |  | <0.001 |
| Leoceed 16 N | 12(19.7) | 9(25.7) | 3(10.0) |  |
| Pureflux 150 H | 21(34.4) | 21(60) | 0(0) |  |
| B-16 H | 11(18) | 0(0) | 11(42.3) |  |
| Elision 210 M | 7(11.5) | 4(11.4) | 3(11.5) |  |
| Revaclear 300 | 6(9.8)b | 0(0.0) | 6(23.1) |  |
| Polyflux 170H | 4(6.6) | 1(2.9) | 3(11.5) |  |
| Dialysis modality (HD/HDF, HD%) | 59(96.7) | 35(100) | 24(92.3) | 0.098 |
| Effective dialysis time(h, mean±SD) | 4.08±0.2 | 4.1±0.2 | 4.0±0.2 | 0.11 |
| Ultrafiltration (L; median, IQR) | 1.6(0.8,2.4) | 1.6(0.0,2.3) | 1.7(1.2,2.5) | 0.61 |
| Residual urine (ml; median, IQR) | 800(350,1500) | 800(400,1300) | 1000(0,1675) | 0.91 |
| Dialysate bicarbonate  (mmol/L, mean±SD) | 32.1±1.8 | 31.8±1.4 | 32.4±2.2 | 0.21 |
| Dialysate potassium  (mmol/L, mean±SD) | 2.8±0.8 | 2.9±0.8 | 2.6±0.7 | 0.18 |
| Dialysate sodium  (mmol/L, mean±SD) | 137.9±1.9 | 137.7±2.3 | 138.1±1.2 | 0.50 |
| Hemoglobin(g/L) | 11.9±1.6 | 12.0±1.4 | 11.6±1.8 | 0.29 |
| Hematocrit (%) | 36.4±5.0 | 37.3±4.4 | 35.1±5.5 | 0.09 |
| Platelets(10^9^/L) | 206±66.4 | 195.0±62.1 | 220.1±70.2 | 0.13 |
| Leukocyte(10^9^/L) | 7.2±2.1 | 6.7±2.0 | 7.8±2.1 | 0.06 |
| Albumin(g/dl) | 4.1±0.4 | 4.1±0.3 | 4.1±0.5 | 0.55 |
| GPT(U/l) | 20.2±12.2 | 18.6±9.1 | 22.4±15.6 | 0.24 |
| GOT(U/l) | 22.9±7.6 | 23.1±8.5 | 22.7±6.3 | 0.86 |
| CRP (g/L) | 12.9±24.8 | 13.5±30.6 | 11.9±13.6 | 0.81 |

Abbreviations: hemodialysis (HD); hemofiltration (HDF). Glutamic pyruvic transaminase (GPT); glutamic oxaloacetic transaminase (GOT); C-reactive protein (CRP). As appropriate, values are reported as either mean ±standard deviation or number (percentage) or median (IQR). Independent samples t-test and Wilcoxon-Mann Whitney U test were used for comparing two units at baseline as applicable. P < 0.05 was considered significant.

**Supplementary table 3**: Changes of dialysis prescription, filters and dialysis modality per treatment period (acetate vs. citrate)

| **Parameter** | **Acetate** | **Citrate** | **P-value** |
| --- | --- | --- | --- |
| **Membranes (n%)** | 61(100%) | 61(100%) | **<0.001** |
| - Leoceed 16 N | 10(16.4) | 6(9.8) |  |
| - Polyflux 150 H | 22(36.1) | 9(14.8) |  |
| - Polyflux 170H | 4(6.6) | 11(18) |  |
| - Polyflux 190H | 2(3.3) | 1(1.6) |  |
| - Polyflux 210H | 0 | 1(1.6) |  |
| - B-16 H | 4(6.6) | 12(19.7) |  |
| - ELISIO 21 H | 6(9.8) | 3(4.9) |  |
| - ELISIO 13 M | 0 | 1(1.6) |  |
| - Revaclear 300 | 13(21.3) | 16(26.2) |  |
| - Other | 0 | 1(1.6) |  |
| **Dialysis modality (HD/HDF, %)** | 61(100%) | 61(100%) | 1.0 |
| - HD | 59(96.7) | 59(96.7) |  |
| - HDF | 2(3.3) | 2(3.3) |  |
| **Vascular access(n%)** | 61(100%) | 61(100%) | 0.79 |
| - AVF | 53(86.9) | 52(85.2) |  |
| - AVG | 3(4.9) | 3(4.9) |  |
| - Catheter | 5(8.2) | 6(9.8) |  |
| **Anticoagulation (n%)** | 61(100%) | 61(100%) | 0.66 |
| - Heparin | 46(75.4) | 48(78.7) |  |
| - Citrate | 1(1.6) | 1(1.6) |  |
| - Low molecular heparin | 14(23) | 12(19.7) |  |
| **Effective session duration**  (h, mean±SD;n=61) | 4.1±0.2 | 3.9±0.3 | 0.039 |
| Residual urine  (ml; mean±SD; n=55 ) | 1079±792 | 1058±811 | 0.22 |
| Effective dialysis time  (h, mean±SD; n=61) | 3.9±0.3 | 4.1±0.2 | **<0.001** |
| Dialysate bicarbonate  (mmol/L, mean±SD; n=61) | 32.1±1.65 | 32.2±1.65 | 0.54 |
| Dialysate potassium  (mmol/L, mean±SD; n=61) | 2.8±0.78 | 2.8±0.79 | 0.71 |
| Dialysate sodium  (mmol/L, mean±SD; n=61) | 138.0±1.9 | 138.3±1.7 | 0.18 |
| Kt/V (n=54) | 1.18±0.29 | 1.19±0.28 | 0.71 |

Abbreviations: hemodialysis (HD); hemodiafiltration (HDF). AVF: Arterio-Venous Fistula; AVG: Arterio-Venous Graft;Kt/V: Dialyzer clearance of urea multiplied by dialysis time and normalized for urea distribution volume, reflecting dialysis adequacy. As appropriate, values are reported as either mean ± standard deviation or count (percentage). Paired samples t-test and Wilcoxon-Mann Whitney U test were used to compare parameters of dialysis prescription pre (Acetate) and post (Citrate buffered A concentrates) Citrate acidified A concentrates treatment as applicable. P < 0.05 was considered significant. Singinificant parameters access type and HD(F) were chosen to adjust the linear mixed effect models as reported in supplementary table 4.

**Supplementary Table 4:** Use of dialysis membranes during acetate and citrate-A-concentrates

| **Acetate** | | **Citrate** | |
| --- | --- | --- | --- |
| **Membranes (n%)** | 61(100%) | **Membranes (n%)** | **61(100%)** |
| Leoceed 16 N | 10(16.4) | Leoceed 16 N | 5(8.2) |
|  |  | ELISIO 21 H | 2(3.3) |
|  |  | B - 16 H | 2(3.3) |
|  |  | Polyflux 170H | 1(1.6) |
| Polyflux 150 H | 22(36.1) | Polyflux 150 H | 9(14.7) |
|  |  | Leoceed 16 N | 1(1.6) |
|  |  | B - 16 H | 10(16.4) |
|  |  | Polyflux 170H | 1(1.6) |
|  |  | ELISIO 13M | 1(1.6) |
| Polyflux 170H | 4(6.6) | Polyflux 170H | 4(6.6) |
| Polyflux 190H | 2(3.3) | Polyflux 190H | 2(3.3) |
| Polyflux 210H | 0 | / | / |
| B-16 H | 4(6.6) | Revaclear 300 | 4(6.6) |
| ELISIO 21 H | 6(9.8) | ELISIO 21 H | 1(1.6) |
|  |  | Polyflux 170H | 3(4.9) |
|  |  | Polyflux 210H | 1(1.6) |
|  |  | Polyflux 190H | 1(1.6) |
| 4ELISIO 13 M | 0 | / | / |
| Revaclear 300 | 13(21.3) | Revaclear 300 | 12(19.7) |
|  |  | others | 1(1.6) |

The table reports counts (percentage) to show the changes of membranes from pre- to post- Citrate A concentrates treatment.

**Supplementary Table 5:** Linear mixed model analysis, adjusted for changes in filters, session duration and dialysis modality per treatment period with treatment as a main effect and cellular phenotypes as dependend variables

| Phenotypes  (109/L)  =  Dependent variable | Linear mixed model: multivariate main effect | | | | | | | | | |
| --- | --- | --- | --- | --- | --- | --- | --- | --- | --- | --- |
|  | Membranes | | Modalities | | Vascular access  AVF = 1 | | Effective session duration | | treatment | |
|  | Estimate (β) | P-value | Estimate (β) | P-value | Estimate (β) | P-value | Estimate (β) | P-value | Estimate (β) | P-value |
| Lymphocyte | -0.80 | 0.79 | 1.1 | 0.33 | -1.1 | 0.13 | -0.3 | 0.49 | 0.27 | 0.19 |
| CD3+CD4+CD69+ | -0.14 | <0.001 | 0.006 | 0.63 | 0.004 | 0.56 | -0.010 | 0.15 | -0.018 | 0.02 |
| CD3+CD8+CD69+ | -0.14 | 0.058 | -0.05 | 0.13 | 0.02 | 0.39 | -0.04 | 0.05 | -0.02 | 0.02 |
| CD14+CD16+PDL1+*^a^* | -0.001 | 0.95 | 0.0006 | 0.68 | <0.0001 | 0.99 | 0.0002 | 0.80 | -0.0007 | 0.12 |
| Leucocyte | 0.37 | 0.39 | 0.99 | 0.51 | -0.93 | 0.46 | -0.68 | 0.2 | -0.036 | 0.87 |
| CD3+ | -1.1 | 0.13 | 1.5 | 0.09 | -0.24 | 0.87 | -0.52 | 0.24 | -0.15 | 0.46 |
| CD3+CD4+ | -0.44 | 0.37 | 1.3 | 0.056 | -0.35 | 0.46 | -0.38 | 0.27 | -0.12 | 0.44 |
| CD3+CD4+CD28+ | -0.29 | 0.40 | 1.3 | 0.06 | -0.37 | 0.4 | -0.38 | 0.27 | -0.11 | 0.48 |
| CD3+CD4+PD1+ | -0.4 | 0.31 | 0.2 | 0.32 | -0.20 | 0.27 | 0.20 | 0.09 | 0.02 | 0.66 |
| CD3+CD4+TIM3+ | -0.05 | 0.20 | 0.02 | 0.16 | -0.04 | 0.72 | -0.0003 | 0.97 | 0.002 | 0.63 |
| CD3+CD8+ | -0.36 | 0.050 | 0.11 | 0.76 | 0.14 | 0.46 | -0.11 | 0.31 | 0.04 | 0.34 |
| CD3+CD8+CD28+ | 0.16 | 0.08 | 0.15 | 0.35 | 0.04 | 0.88 | -0.01 | 0.83 | 0.01 | 0.63 |
| CD3+CD8+PD1+ | -0.29 | 0.068 | -0.07 | 0.63 | 0.05 | 0.49 | 0.12 | 0.041 | -0.02 | 0.43 |
| CD3+CD8+TIM3+ | -0.009 | 0.52 | -0.008 | 0.04 | <0.0001 | 0.96 | -0.003 | 0.18 | -0.001 | 0.15 |
| CD4/CD8 | 1.2 | 0.74 | 6.0 | 0.08 | -1.3 | 0.032 | -0.16 | 0.79 | 0.02 | 0.94 |
| Monocyte | 0.89 | 0.33 | 0.03 | 0.97 | -0.15 | 0.71 | 0.18 | 0.65 | -0.02 | 0.91 |
| CD14+CD16- *^a^* | 0.65 | 0.54 | 0.17 | 0.72 | -0.002 | 0.83 | 0.11 | 0.72 | -0.11 | 0.43 |
| CD14+CD16+ *^a^* | -0.03 | 0.95 | 0.01 | 0.90 | 0.06 | 0.49 | 0.04 | 0.43 | -0.12 | 0.69 |
| CD14-CD15+  PMN-MDSC *^b^* | -0.03 | 0.52 | 0.01 | 0.65 | 0.02 | 0.51 | -0.006 | 0.69 | -0.01 | 0.36 |
| CD14+CD16-PDL1+ *^a^* | 0.008 | 0.74 | 0.003 | 0.68 | -0.004 | 0.50 | -0.001 | 0.81 | -0.003 | 0.17 |

Linear mixed models were built to analyze the effect of hemodialysis prescription on cellular phenotypes (dependent variable) before and after switching to citrate dialysate (treatment Citrate=1). These models were adjusted for: membrane type used, HD(F)-modalites,vascular access, and session duration, to test for robustness of our results and exclude confounding by these parameters. P <0.05 was considered significant. a.1 missing values in the citrate treatment group. b. 9 missing values of PMN-MDSCs in acetate treatment groups.

**Supplementary Table 6:** Linear mixed model analysis, adjusted for changes in filtration Volume, Immunosupression medication and dialysis modality per treatment period with treatment as a main effect and cellular phenotypes as dependend variables.

| Phenotypes  (109/L)  =  Dependent variable | Linear mixed model: multivariate main effect | | | | | | | |
| --- | --- | --- | --- | --- | --- | --- | --- | --- |
|  | Filtration Volume | | Modalities | | Immunosuprression  Medication | | treatment | |
|  | Estimate (β) | P-value | Estimate (β) | P-value | Estimate (β) | P-value | Estimate (β) | P-value |
| Lymphocyte | -1.5 | 0.51 | 0.9 | 0.36 | 0.49 | 1.95 | 0.0.29 | 0.12 |
| CD3+CD4+CD69+ | -0.007 | 0.27 | 0.004 | 0.65 | 0.001 | 0.78 | -0.018 | 0.02 |
| CD3+CD8+CD69+ | -0.01 | 0.33 | -0.01 | 0.62 | 0.01 | 0.41 | -0.008 | 0.37 |
| CD14+CD16+PDL1+*^a^* | <0.001 | 0.65 | 0.001 | 0.367 | 0.0002 | 0.61 | -0.0009 | 0.026 |
| Leucocyte | -1.76 | 0.45 | 1.49 | 2.74 | 0.58 | 0.26 | -0.02 | 0.91 |
| CD3+ | -2.0 | 0.096 | 1.3 | 0.73 | -0.18 | 0.5 | -0.1 | 0.6 |
| CD3+CD4+ | -1.1 | 0.12 | 1.1 | 0.039 | 0.038 | 0.85 | -0.14 | 0.32 |
| CD3+CD4+CD28+ | -1.1 | 0.13 | 1.1 | 0.05 | 0.038 | 0.85 | -0.14 | 0.32 |
| CD3+CD4+PD1+ | -0.4 | 0.31 | 0.2 | 0.32 | -0.20 | 0.27 | 0.02 | 0.66 |
| CD3+CD4+TIM3+ | -0.01 | 0.08 | 0.014 | 0.27 | -0.002 | 0.73 | 0.003 | 0.44 |
| CD3+CD8+ | -0.83 | 0.067 | 0.14 | 0.64 | 0.15 | 0.18 | 0.014 | 0.76 |
| CD3+CD8+CD28+ | -0.54 | <0.01 | 0.13 | 0.32 | 0.17 | 0.044 | -0.003 | 0.89 |
| CD3+CD8+PD1+ | -1.4 | 0.30 | -0.13 | 0.92 | 0.057 | 0.25 | 0.015 | 0.49 |
| CD3+CD8+TIM3+ | -0.007 | 0.15 | -0.006 | 0.04 | 0.002 | 0.11 | <-0.001 | 0.51 |
| CD4/CD8 | 2.8 | 0.1.93 | 3.9 | 1.4 | -0.87 | 0.38 | -0.18 | 0.31 |
| Monocyte | -0.14 | 0.35 | 0.63 | 0.26 | 0.29 | 0.18 | -0.17 | 0.27 |
| CD14+CD16- *^a^* | -0.11 | 0.46 | 0.61 | 0.1.4 | 0.31 | 0.55 | -0.20 | 0.11 |
| CD14+CD16+ *^a^* | 0.03 | 0.90 | 0.07 | 0.38 | 0.03 | 0.26 | -0.004 | 0.89 |
| CD14-CD15+  PMN-MDSC *^b^* | -0.01 | 0.82 | 0.02 | 0.39 | 0.02 | 0.042 | -0.02 | 0.25 |
| CD14+CD16-PDL1+ *^a^* | <0.001 | 0.83 | 0.01 | 0.13 | 0.002 | 0.50 | -0.004 | 0.10 |

Linear mixed models were built to analyze the effect of hemodialysis prescription and immunosuppression medication on cellular phenotypes (dependent variable) before and after switching to citrate dialysate (treatment Citrate=1). These models were adjusted for: filtration Volume, membrane type used, HD(F)-modalites, immuno- suppression medication, to test for robustness of our results and exclude confounding by these parameters. P <0.05 was considered significant. a.1 missing values in the citrate treatment group. b. 9 missing values of PMN-MDSCs in acetate treatment group
