# Supplementary material for: Immunophenotypic Characterization of Citrate-Containing A Concentrates in Maintenance Hemodialysis: A Pre-Post Study
Source: Int J Nephrol. 2023 Sep 27;2023:7772677. doi: 10.1155/2023/7772677 (PMC10551471; doi:10.1155/2023/7772677)
Supplement: Supplementary Materials — Supplementary Figure 1: A–N: the gating ancestry of T cells from peripheral blood mononuclear cells isolated by CPT® gradient centrifugation ex vivo. Cells were gated as displayed in A–E to isolate CD3+ cells. F–J: applied for surface marker gating on CD3+CD4+cells. A similar approach was applied for CD3+CD8+ cells (K–N). Supplementary Figure 2: the gating strategy of monocytes from peripheral blood mononuclear cells isolated by CPT® gradient centrifugation ex vivo. A–H: selection of CD14+/CD16 ± monocytes. I–J: defining PDL+1 cells amongst CD14+CD16 ± cells, respectively. Supplementary Figure 3: A–F: gating strategy of MDSCs from peripheral blood mononuclear cells isolated by CPT® gradient centrifugation ex vivo. Supplementary Figure 4: alterations of T-cell exhaustion markers before and after undergoing acetate or citrate-buffered A concentrates for three months, respectively. The number of phenotypes is presented as histograms (mean and standard error of mean) for 61 patients. Level of significance P < 0.05. Supplementary Table 1: monoclonal antibodies used for flow cytometry. Supplementary Table 2: dialysis characteristics and dialysate compositions at baseline. Supplementary Table 3: changes of dialysis prescription, filters, and dialysis modality per treatment period (acetate vs. citrate). Supplementary Table 4: use of dialysis membranes during acetate and citrate A concentrates. Supplementary Table 5: linear mixed model analysis, adjusted for changes in filters, session duration, and dialysis modality per treatment period with treatment as a main effect and cellular phenotypes as dependent variables. Supplementary Table 6: linear mixed model analysis, adjusted for changes in filtration volume, immunosupression medication, and dialysis modality per treatment period with treatment as a main effect and cellular phenotypes as dependent variables. [file 7772677.f1.zip › supplementary_only_figures.docx]

**Supplementary figures: Immunophenotypic characterization of citrate containing A concentrates in maintenance hemodialysis – a pre-post study.**

**Supplementary Figure 1**


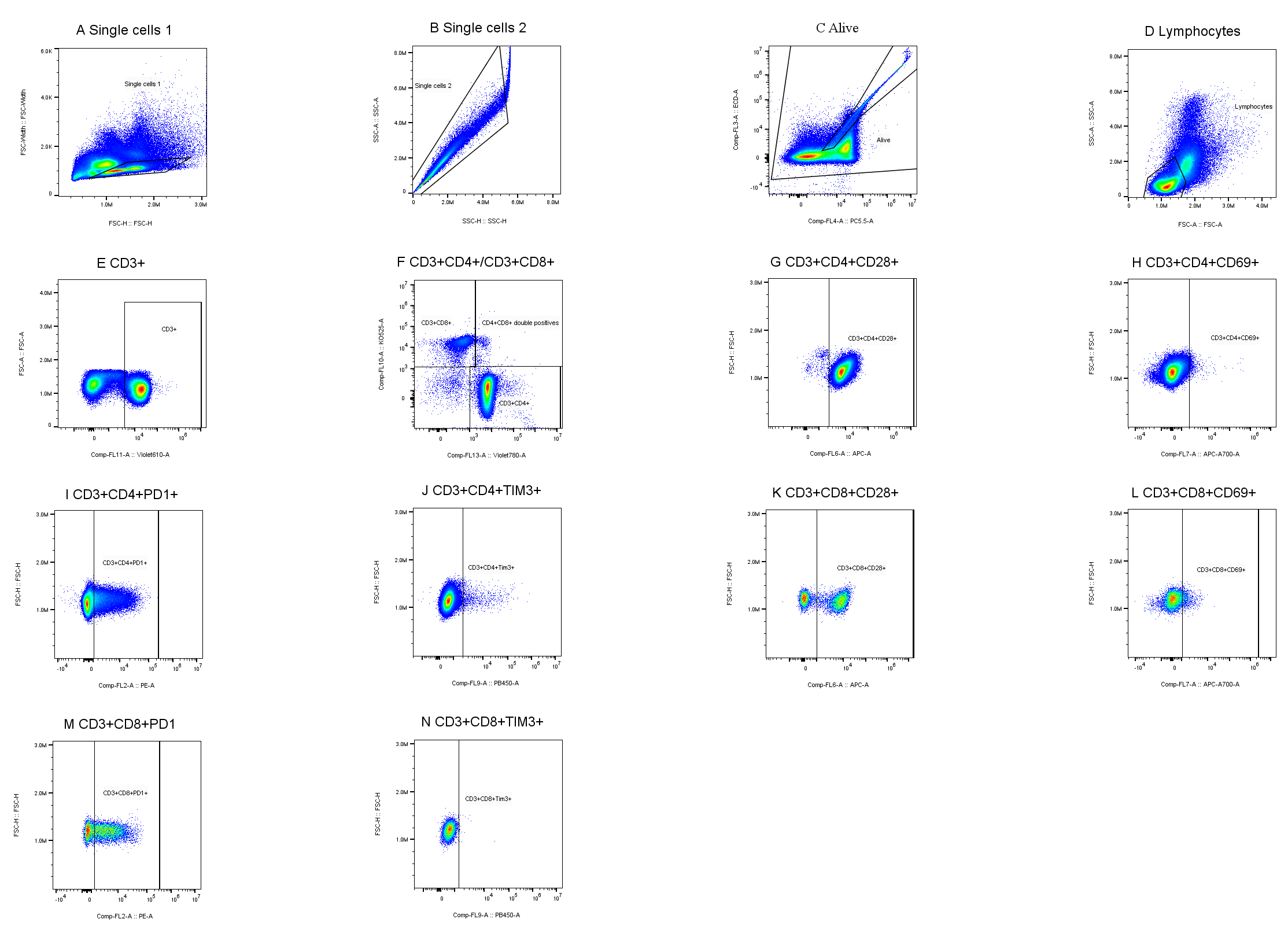


**Supplementary Fig.1.** A-N reports the gating ancestry of T cells from peripheral blood mononuclear cells isolated by CPT® gradient centrifugation ex vivo. Cells were gated as displayed in A-E to isolate CD3+ cells. F to J was applied for surface marker gating on CD3+CD4+cells. A similar approach was applied for CD3+CD8+ cells (K-N).

**Supplementary Figure 2**


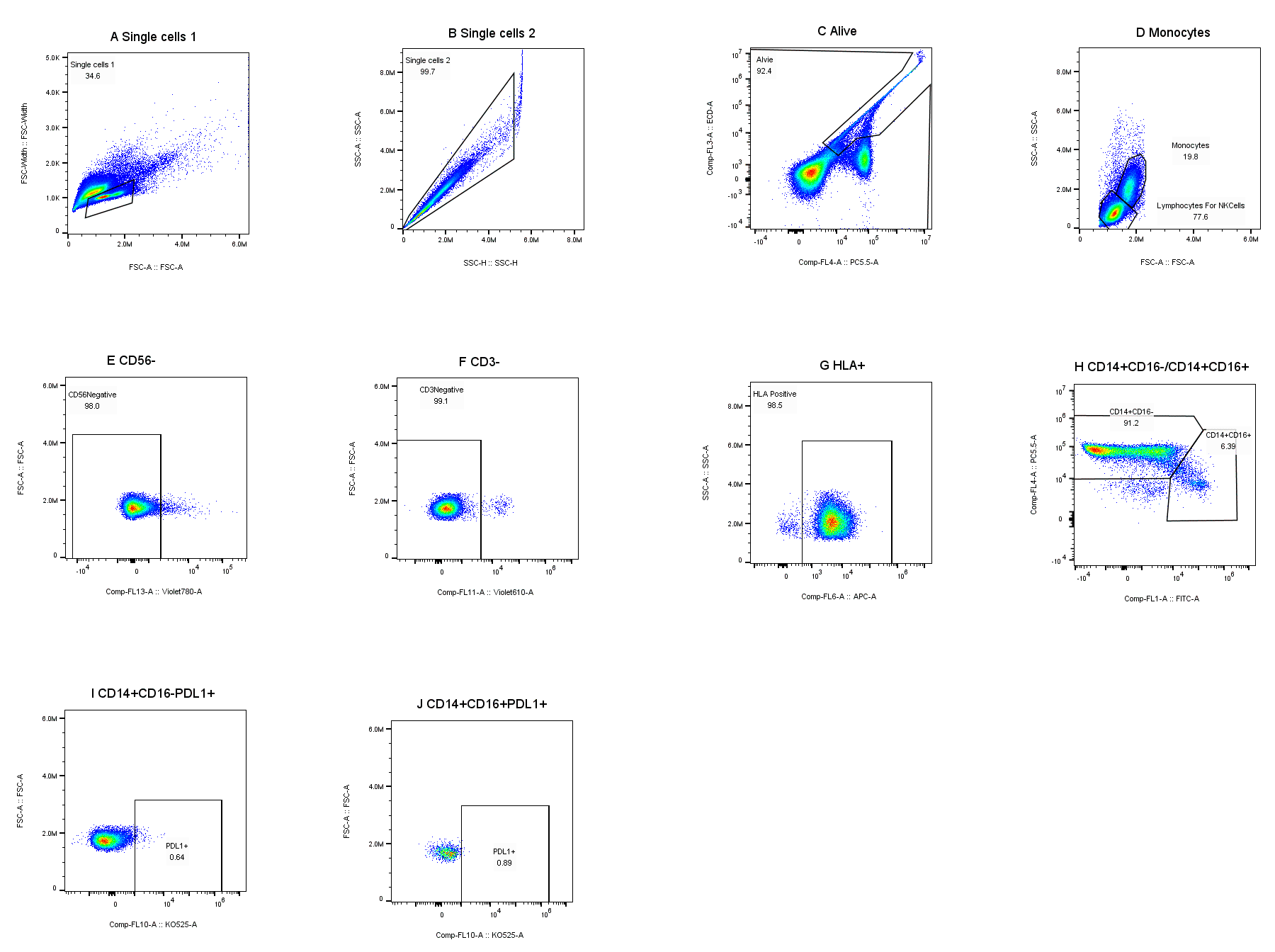


**Supplementary Fig.2.** Displays the gating strategy of Monocytes from peripheral blood mononuclear cells isolated by CPT® gradient centrifugation ex vivo. A to H were used to select CD14+/CD16+/- monocytes. I and J were used to define PDL+1 cells amongst CD14+CD16+/- cells, respectively.

**Supplementary Figure 3**


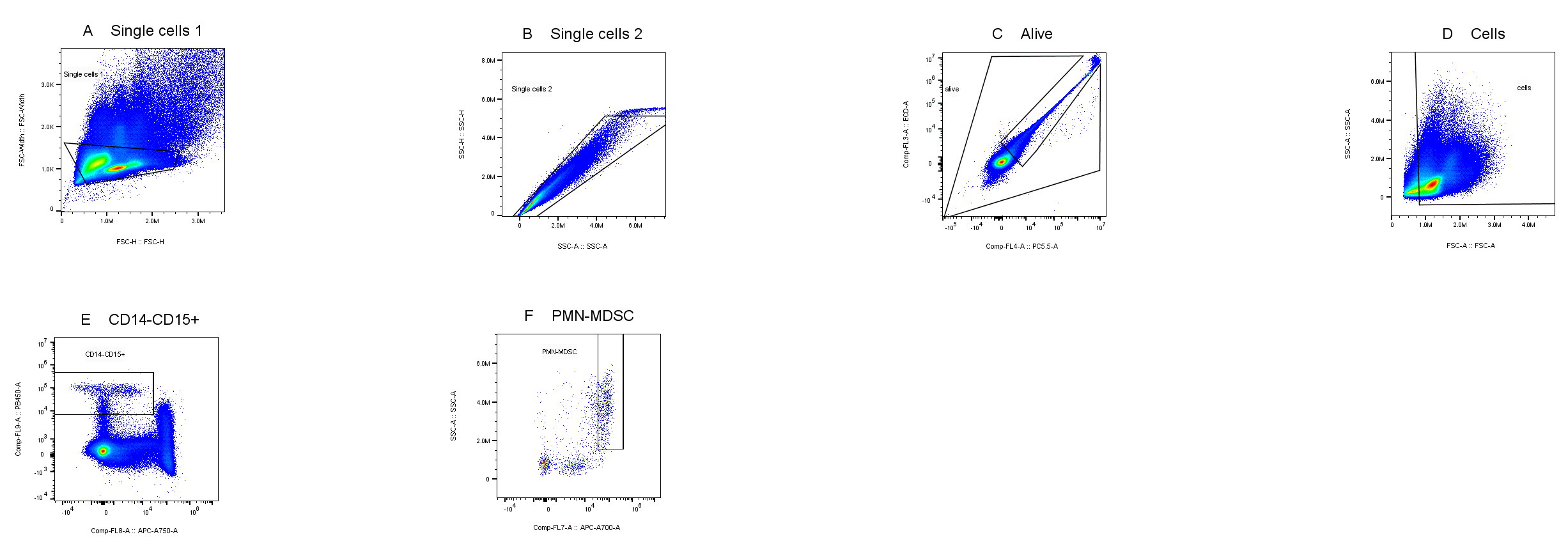


**Supplementary Fig.3.** A-F: Gating strategy of MDSCs from peripheral blood mononuclear cells isolated by CPT® gradient centrifugation ex vivo.

**Supplementary Figure 4**


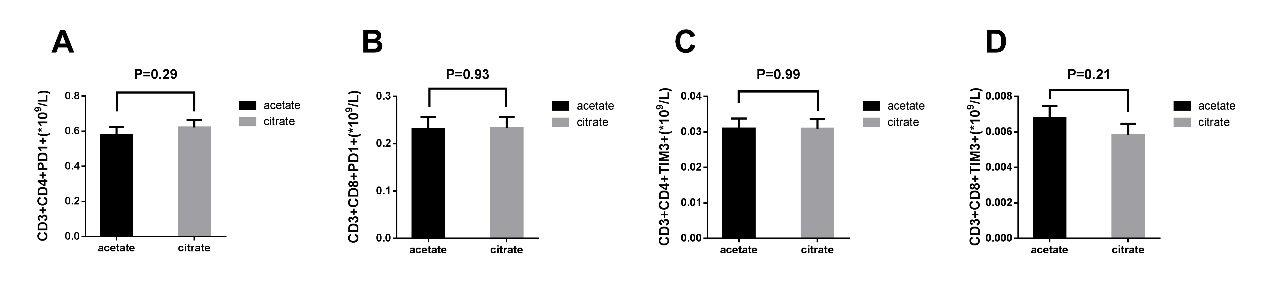


**Supplementary Fig.4.**Displays alterations of T cell exhaustion markers before and after undergoing acetate or citrate buffered A concentrates for three months, respectively. The number of phenotypes is presented as histograms (mean and standard error of mean) for 61 patients. Level of significance P<0.05.
